# The High Prevalence of Hypovitaminosis D in China: A Multicenter Vitamin D Status Survey

**DOI:** 10.1097/MD.0000000000000585

**Published:** 2015-02-27

**Authors:** Songlin Yu, Huiling Fang, Jianhua Han, Xinqi Cheng, Liangyu Xia, Shijun Li, Min Liu, Zhihua Tao, Liang Wang, Li’an Hou, Xuzhen Qin, Pengchang Li, Ruiping Zhang, Wei Su, Ling Qiu

**Affiliations:** From the Department of Clinical Laboratory (SY, HF, JH, XC, LX, LH, XQ, PL, WS, LQ), Peking Union Medical College Hospital, Chinese Academy of Medical Sciences, Beijing; Department of Clinical Laboratory (SL), The First Affiliated Hospital of Dalian Medical University, Dalian; Department of Clinical Laboratory (ML), The First Affiliated Hospital, Sun Yat-sen University, Guangdong; Department of Clinical Laboratory (ZT), The Second Affiliated Hospital of Zhejiang University School of Medicine, Hangzhou; Department of Clinical Laboratory (LW), Xinjiang Medical University, Xinjiang; and Department of Clinical Laboratory (RZ), China–Japan Friendship Hospital, Beijing, China.

## Abstract

Vitamin D deficiency, which is usually detected by using immunoassays or the more reliable liquid chromatography tandem mass spectrometry (LC-MS/MS) methods, has recently been considered a public health problem worldwide. However, the vitamin D status in Chinese populations, as measured using the LC-MS/MS method, is not available. The objective of this multicenter study was to determine the vitamin D status and prevalence of vitamin D deficiency by using a reliable method in 5 large cities in China.

From May 1 to September 31, 2013, we conducted a multicenter study on 2173 apparently healthy adults who were recruited from 5 Chinese cities. The 25-hydroxyvitamin D 25OHD_2_ and 25OHD_3_ levels were measured using the LC-MS/MS method. Intact parathyroid hormone (iPTH), calcium, phosphorus, and alkaline phosphate levels were also measured using an automatic analyzer.

The mean 25OHD level of all participants was 19.4 ± 6.4 ng/mL (2.5–97.5%: 7.9–32.6 ng/mL), and only 109 (5.0%) participants had a 25OHD_2_ level >2.5 ng/mL (maximum, 22.4 ng/mL). In this study, the prevalence of severe vitamin D deficiency (<10 ng/mL), vitamin D deficiency (10–20 ng/mL), vitamin D insufficiency (20–30 ng/mL), and vitamin D sufficiency (>30 ng/mL) was 5.9%, 50.0%, 38.7%, and 5.4%, respectively. Women had a significant higher rate of deficiency than men (66.3% vs 45.3%, *P *< 0.01). Participants aged 18 to 39 years had a lower 25OHD level than elderly individuals (>59 years). Lifestyle may influence the 25OHD level more than the latitude, with participants in Dalian having the highest 25OHD level and the lowest deficiency rate. The serum iPTH level showed a significant negative correlation with the 25OHD level (*r* = −0.23, *P *< 0.01) after correcting for age and sex.

In conclusion, the present study evaluated the vitamin D status using a reliable method, and our results indicate that vitamin D deficiency is prevalent among all age groups in China, especially among younger adults. We also observed significant differences in the 25OHD levels according to sex, age, and region among apparently healthy individuals.

## Abstract 2

Supplemental Digital Content is available in the text

## INTRODUCTION

Vitamin D regulates bone and mineral metabolism by balancing level of calcium (Ca) and phosphorus (P). Recent studies have also reported the association between vitamin D and diabetes,^1^ cardiovascular diseases,^2,3^ and cancer,^4^ and these results have revived interest in this important hormone. Numerous studies have conducted previously to assess vitamin D status in most countries and continents, and these have reported that vitamin D deficiency is common, particularly among young children, pregnant women, and the elderly.^5,6^

To our knowledge, no Chinese studies have thus far evaluated a large population of apparently healthy adults, as most research has focused on special age groups with or without diseases. For example, Foo et al^7^ reported vitamin D data for girls aged 12 to 14 years in Beijing, whereas Lu et al^8^ reported the association of vitamin D status with metabolic syndrome among middle-aged and elderly Chinese individuals. Another report^9^ described the vitamin D status in Shanghai using data collected from several community centers. However, vitamin D levels are dependent on various factors including age, sunshine exposure, clothing habit, skin pigmentation, use of sun blocks, nutrition, and supplements.^10^ Therefore, data from specific age groups or a single province cannot accurately reflect the general vitamin D status of the Chinese population.

25-Hydroxyvitamin D (25OHD) is currently considered the most accurate biomarker of serum vitamin D levels and is derived from cutaneous synthesis and dietary intake.^10,11^ Immunoassay is the most commonly used method for evaluating the 25OHD level, although it may be limited by antibody cross-reactivity, nonequimolar detection of 25OHD_2_ and 25OHD_3_, and high interlaboratory variation (up to 38%).^12–14^ Researchers have attempted to determine an optimum cutoff value for vitamin D deficiency, although there is still tremendous variability regarding the definition of vitamin D deficiency or adequate vitamin D status. However, most investigators agree that a 25OHD level <20 ng/mL can be defined as vitamin D deficiency, although several guidelines recommend even lower cutoffs.^15–17^ The combination of variable immunoassay methods and different definitions of deficiency make interstudy comparisons difficult.

More recently, liquid chromatography tandem mass spectrometry (LC-MS/MS) has become the gold standard for measuring 25OHD levels.^18,19^ LC-MS/MS provides higher specificity, sensitivity, and reproducibility compared with immunoassays, and can quantify 25OHD_2_ and 25OHD_3_ separately.^20^ Unfortunately, the early studies of vitamin D status in China used the immunoassay method,^7–9^ which might not accurately estimate the vitamin D deficiency prevalence, and did not evaluate 25OHD_2_ levels.

Given the important role of vitamin D in bone health and nonskeletal diseases, there is an urgent need for accurate data regarding the vitamin D status in Chinese population. Thus, we conducted this multicenter study with participants from 5 provinces in China, and evaluated all samples at a central laboratory with a reliable LC-MS/MS assay to enhance future comparison.

## METHODS

### Study Population

The study population was a part of the International Federation of Clinical Chemistry (IFCC) Global Multicenter study of reference intervals in China. According to the IFCC Committee on Reference Intervals and Decision Limits protocol,^21^ 2627 healthy volunteers were recruited from 5 representative geographical cities in China—Dalian (northeast), Beijing (north), Hangzhou (east), Guangzhou (south), and Urumqi (northwest)—between May 1 and September 30, 2013. The participants were invited by posters or orally to the physical examination centers of the following 5 hospitals, each in 1 of the 5 cities, respectively: Peking Union Medical College Hospital, the First Affiliated Hospital of Dalian Medical University, the First Affiliated Hospital, of Sun Yat-sen University, The Second Affiliated Hospital of Zhejiang University School of Medicine, and Xinjiang Medical University. All the participants were explained the including/exclusion criteria of the study, the consent form, and procedures for participation. The main target range of ages was 18 to 65 years. However, individuals aged >65 years were also included if they matched the inclusion criteria. An even distribution of sex and age was ensured by tabulating volunteers.^21^ This study design was approved by the Ethics Committee of Peking Union Medical College Hospital.

Inclusion criteria were set a priori as living in the selected region for >1 year; free from acute or chronic infections, digestive diseases, kidney disease, metabolic and nutritional disease, rheumatic diseases, endocrine disease, or circulation system diseases; and had not undergone surgery, or received medication, blood donation, or transfusion within the previous 6 months. Participants who were currently taking vitamin D supplements were excluded. Height and weight were measured by a trained research nurse, and body mass index (BMI) was calculated as weight divided by height squared. Information related to the above inclusion and exclusion criteria as well as the baseline subject characteristics were obtained using self-administered questionnaires.

### Laboratory Measurements

One week before sample collection, patients were asked to maintain their normal dietary intake, although they were requested to avoid alcohol intake (48 hours) and smoking (1 hour) before the collection. Participants were asked to refrain from eating 12 hours before the sampling. Fasting blood samples were taken by venipuncture into tubes containing procoagulant, centrifuged (1200× *g*, 10 minutes) within 2 hours, aliquoted, and frozen at −80°C. The frozen serum samples were then transported on dry ice to the laboratory at Peking Union Medical Hospital, and were stored at −80°C until analysis.

Serum 25OHD_2_ and 25OHD_3_ levels were measured with a modified LC-MS/MS method.^20^ Briefly, 0.2-mL aliquots of calibrators or serum samples were mixed with 0.2 mL of the isotope-labeled internal standard solution in methanol and then treated with 0.1 mmol/L sodium hydroxide, precipitated with zinc sulfate solution. The 25OHD was then extracted with 1 mL hexane. After centrifugation, 0.8 mL supernatant was transferred and was evaporated under a stream of nitrogen until dryness and reconstituted with 0.2 mL methanol: water (80:20). Subsequently, the residuals were analyzed using the LC-MS/MS system. Chromatographic separation was performed using a Waters ACQUITY UPLC BEH (Waters Technologies (Shanghai) Limited. Beijing Co. Shanghai, China) Phenyl column (2.1 × 100 mm, 1.7 μm) with mobile phase A comprising methanol and phase B comprising water with 0.1% formic acid. The isocratic gradient was as follows: 0 to 0.1 minute 60% A, 0.1 to 2 minutes 75% A, 2 to 3 minutes 98% A, 3 to 3.1 minutes 60% A, and 3.1 to 4.5 minutes 60% A, at a flow rate of 0.4 mL/min. An API 4000 QTRAP triple quadruple mass spectrometer (Sciex Applied Biosystems, Foster City, CA) was used for the MS/MS detection in the positive electrospray ionization mode and multiple reaction monitor (MRM) mode, and the MRM transitions used for each analyte were as follows: m/z 413.3→395.3 [25(OH)D_2_], 401.4→383.4 [25(OH)D_3_], 416.3→398.3 [25(OH)D_2_ internal standard], and 404.4→386.4 [25(OH)D_3_ internal standard]. The representative chromatograph is shown in Supplementary Figure 1, http://links.lww.com/MD/A218. The detection range for both 25OHD_3_ and 25OHD_2_ is 2.5 to 200 ng/mL, with imprecision of 1.89% to 2.67% and 2.58% to 7.90%, respectively. The accuracy of this method was validated by analyzing the National Institute of Standards and Technology SRM 972. Compared with the reference values of SRM 972a, the accuracy of this method was 100.0% to 106.9% and 96.0% to 100.0% for 25OHD_2_ and 25OHD_3_, respectively.

Serum iPTH was measured by electroluminescence immunoassay, using the DXI 800 automatic analyzer (Beckman Coulter, Miami, FL). Serum Ca, P, and alkaline phosphate (ALP) levels were measured enzymatically by using the AU5800 automatic analyzer (Beckman Coulter). All reagents and calibrators were obtained from the corresponding instrument's manufacturer.

### Definitions

We used the commonly accepted cutoffs for severe 25OHD deficiency (<10 ng/mL), deficiency (10–20 ng/mL), and insufficiency (20–30 ng/mL) to stratify our participants. A level of ≥30 ng/mL was considered sufficient or optimum, and a level >150 ng/mL was considered vitamin D intoxication.^10,17^

### Statistical Analysis

Statistical analysis was performed using SPSS 17.0 (SPSS Inc., Chicago, IL). Continuous variables were reported as mean ± standard deviation. Subgroup analysis was conducted according to sex, age, or region, and mean 25OHD concentrations were compared using independent sample *t* tests or 1-way analysis of variance. The prevalence of various 25OHD statuses was compared between regions using Pearson χ^2^ test. Spearman correlation coefficients were calculated for serum 25OHD and iPTH levels, as well as other biochemical markers, after adjusting for age and sex. Differences were considered statistically significant at a *P* value <0.05.

## RESULTS

### Characteristics of the Study Population

After exclusions, 2173 individuals were enrolled and included in our analysis (male:female = 0.98:1). Their demographical information and levels of Ca, P, ALP, and iPTH are presented in Table 1. The mean 25OHD level was 19.4 ± 6.4 ng/mL, with 109 (5.0%) participants having 25OHD_2_ level >2.5 ng/mL. Of these 109 participants, 98 (90.0%) had a level of 2.5 to 10 ng/mL (maximum, 22.4 ng/mL). The distribution of participants with detectable levels of 25OHD_2_ was as follows: 19 (4.6%) in Beijing, 23 (5.3%) in Hangzhou, 54 (12.3%) in Guangzhou, 5 (1.0%) in Dalian, and 8 (2.0%) in Urumqi. No significant difference was observed in the age of participants from the 5 regions, although the iPTH level in Dalian was significantly lower than that in the other 4 regions (*P* value <0.01).

**TABLE 1:**
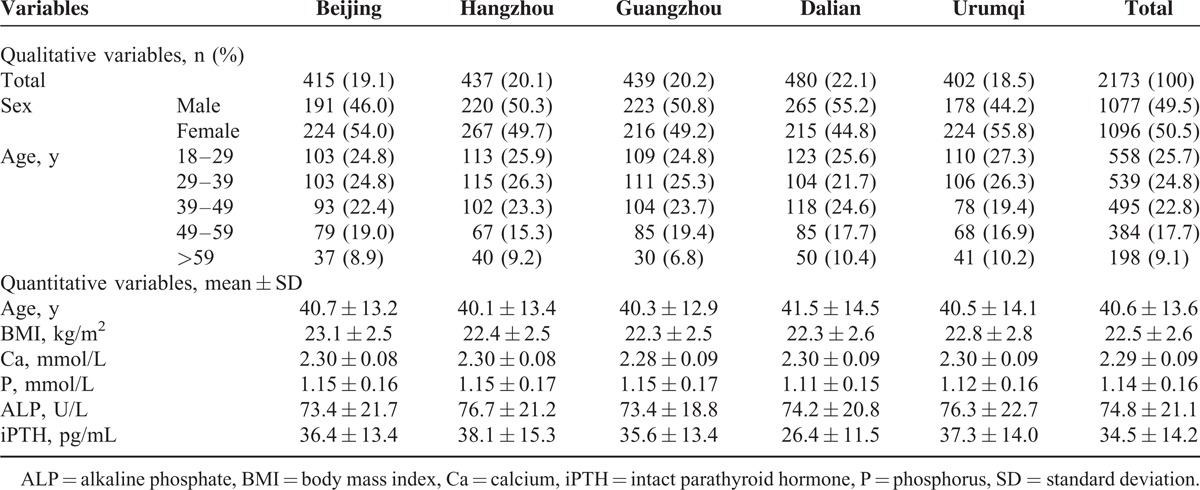
Characteristics of the Study Population

### 25OHD Status and the Prevalence of Deficiency

**FIGURE 1:**
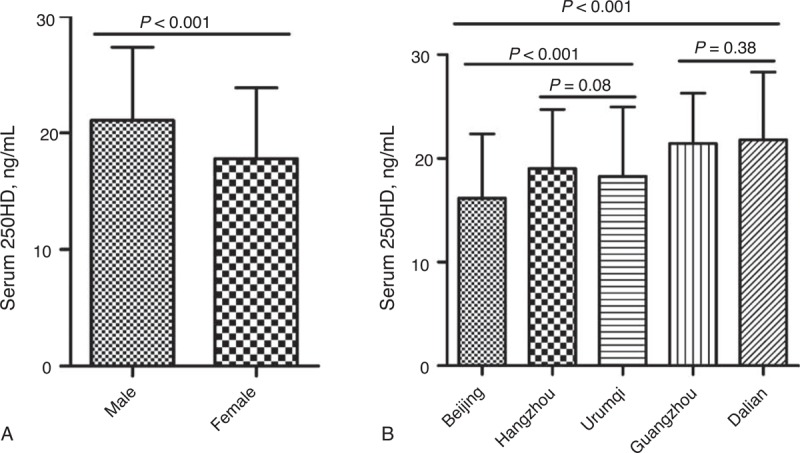
Mean level of serum 25OHD according to subgroup. (A) Male and female. (B) Various regions. Bars indicate stand deviation, and *P* values were calculated using independent 2 sample *t* tests. 25OHD = 25-hydroxyvitamin D.

A significant difference was detected in the 25OHD levels of male and female participants, as well as between the various regions (Figure 1). Therefore, we also separately analyzed the 25OHD status of each region (Table 2). Participants from Dalian (21.8 ± 6.6 ng/mL) and Guangzhou (21.4 ± 4.9 ng/mL) had significantly higher 25OHD levels than those from Hangzhou (19.0 ± 5.7 ng/mL), Urumqi (18.3 ± 6.7 ng/mL), and Beijing (16.2 ± 6.2 ng/mL); the 25OHD status of participants from Beijing was significantly lower than that of participants from the other 4 cities’ (Figure 1). Beijing had the highest rate of 25OHD deficiency (73.5%), followed by Urumqi (66.8%), Hangzhou (60.6%), Dalian (42.1%), and Guangzhou (39.6%). The overall prevalence of 25OHD deficiency in this study was 55.9%, and 94.6% participants exhibited 25OHD deficiency or insufficiency.

**TABLE 2:**
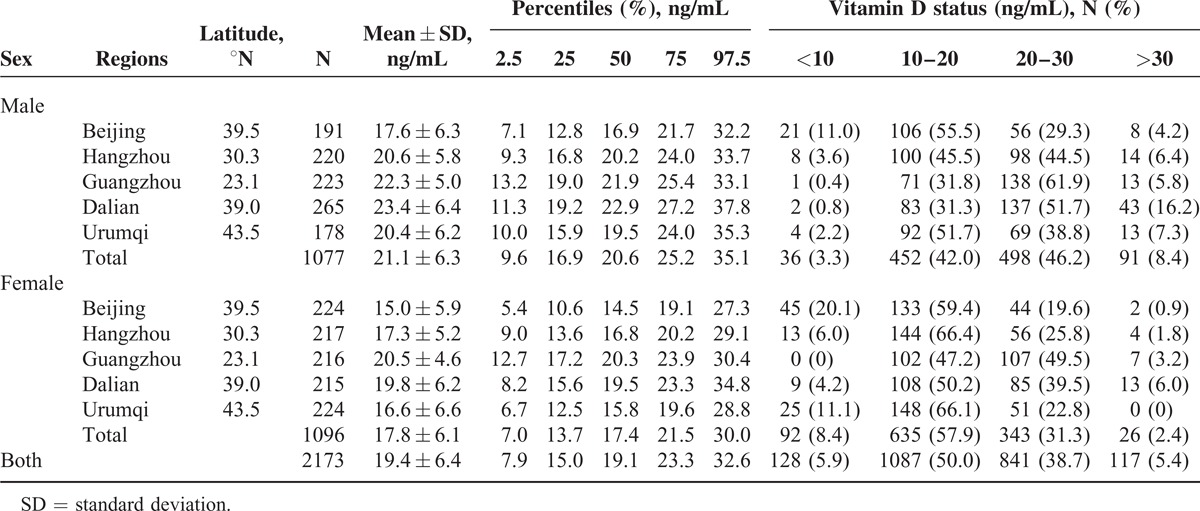
Distribution of 25-hydroxyvitamin D by Region and Sex

### 25OHD Level by Age Group

The results indicate a strong age dependence among 25OHD levels; the 25OHD levels in participants aged 49 to 59 and >59 years were significantly higher than in participants aged 18 to 29 and 29 to 39 years (Supplementary Table 1, http://links.lww.com/MD/A218). However, male residents of Beijing aged 49 to 59 years had significantly lower 25OHD level than those from the other 4 age groups in Beijing. The age dependence among 25OHD levels was slightly different for male and female participants (Supplementary Table 2, http://links.lww.com/MD/A218), although younger adults (18–39 years) generally had lower 25OHD levels than adults >49 years.

### Association Between 25OHD and Other Related Biomarkers

**FIGURE 2:**
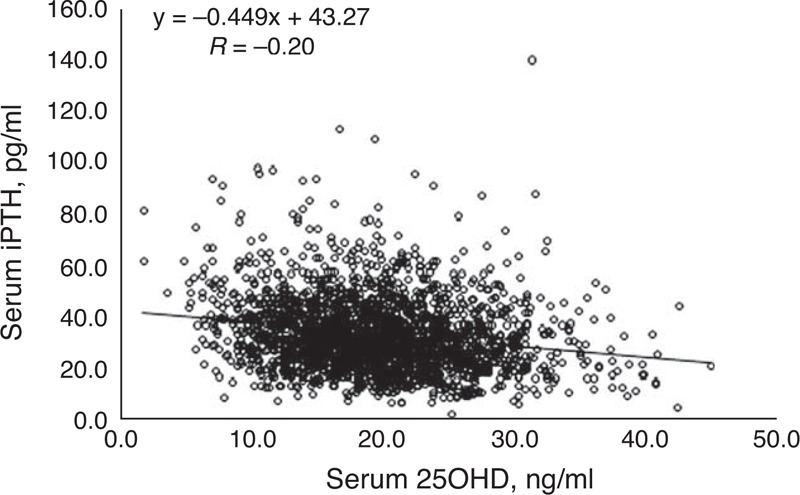
Correlation between level of iPTH and 25OHD. 25OHD = 25-hydroxyvitamin D, iPTH = intact parathyroid hormone.

Significant differences in age, BMI, Ca, P, ALP, and iPTH were detected when participants were stratified by 25OHD status (Table 3). As 25OHD status improved from severe deficiency to sufficiency, BMI and Ca gradually increased, whereas P and iPTH decreased. The correlation between iPTH and 25OHD was significant (*r* = −0.20, *P* < 0.01) (Figure 2). Even after correcting for age and sex, Spearman correlation analysis revealed that a significant positive relationship existed between Ca and 25OHD levels (*r* = 0.08, *P* < 0.01), whereas iPTH negatively correlated with 25OHD (*r* = −0.23, *P *< 0.01). The relationship between iPTH and 25OHD was also observed among the 118 participants with 25OHD level >30 ng/mL.

**TABLE 3:**
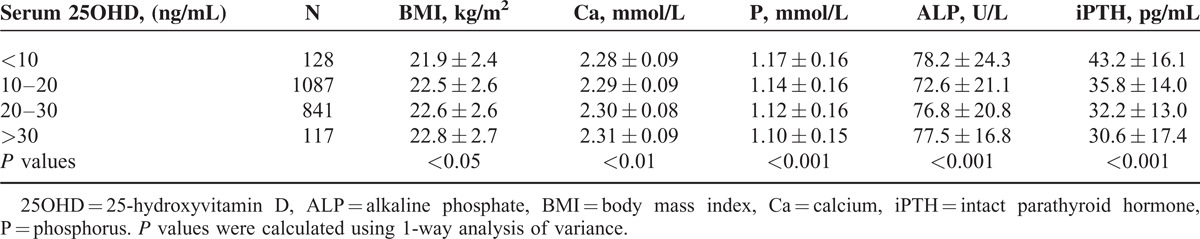
BMI, Serum iPTH, Serum ALP, Serum Ca, and Serum P According to 25OHD Status

## DISCUSSION

Although 25OHD deficiency is common worldwide, national data regarding its prevalence in China does not exist. To our knowledge, this is the first multicenter study to analyze 25OHD status in diverse Chinese cities, with a large study population covering all adult age groups, and using the LC-MS/MS method. One strength of this study is the use of LC-MS/MS, whereas another is single-center analysis by a single experienced technician, which eliminates the possibility of interlaboratory variation. Our findings are consistent with those of previous studies reporting that vitamin D deficiency or insufficiency was more severe in women,^5^ although we did observe a higher prevalence of 25OHD deficiency or insufficiency compared with that in previous studies. Lu et al^9^ have reported that vitamin D insufficiency prevalence was 84% in men and 89% in women, whereas vitamin D deficiency was 30% and 46%, respectively. Zhang et al^22^ also reported that the prevalence of vitamin D insufficiency and sufficiency was 82.4% and 17.6%, respectively. In contrast, we observed a higher prevalence of vitamin D insufficiency (men, 91.6%; women, 92.5%) and deficiency (men, 45.3%; women, 66.3%). These differences may be because 25OHD levels were overestimated due to cross-reaction in the immunoassays used previously.^9,22^ Although these cutoff values are widely used in the literature, they were determined using the immunoassay method, and therefore, future studies should evaluate whether the same cutoff values are applicable to the LC-MS/MS method. The cutoff values for the 2 methods may not be similar; therefore, our comparisons should be interpreted with caution.

Although there are some reports on the vitamin D status in some cities in China,^7–9^ the data from these reports is limited by the immunoassay method used. Furthermore, only the total 25OHD was reported. This study is the first to present data regarding the 25OHD_2_ levels in China. Circulating 25OHD is mainly produced by the skin after exposure to ultraviolet B radiation (25OHD_3_) or is obtained from dietary sources or supplements (both 25OHD_3_ and 25OHD_2_). Biological equivalence of vitamin D_3_ and D_2_ have been controversial.^23,24^ Some studies showed that vitamin D_2_ was much less effective than vitamin D_3_ in humans^23^ whereas others found they are equally effective.^24^ Moreover, nonequimolar recognition of 25OHD_2_ may have caused large variations among immunoassays worldwide.^25^ Therefore, it is important to understand the distributions of vitamin D_2_ status in the Chinese population. By using the improved LC-MS/MS method, measuring vitamin D_2_ levels would be possible. In our study, vitamin D_2_ was detected in an average of 5.0% studied participants; however, the percentage was different among the 5 cities, probably because of differences in climate and lifestyle.

As sunshine is the most important source of vitamin D,^10^ persons living closer to the equator might be expected to have higher vitamin D levels than those living at higher latitudes. However, we did not observe a significant relationship between serum 25OHD levels and regional latitude. For example, Dalian is located north of Guangzhou and Hangzhou, and has a similar latitude to Beijing and Urumqi. However, the 25OHD levels of participants in Dalian were similar to those of participants in Guangzhou and were significantly higher than those of participants in Hangzhou, Urumqi, and Beijing. This result may be related to fish oil consumption, rather than latitude, as both Dalian and Guangzhou are coastal cities where the population consumes large amounts of fish. A positive relationship between northern latitudes and serum 25OHD levels in Europe has been reported,^26^ and Brustad et al^27^ have reported that Norwegian costal populations (latitude, 70°N) consume large amounts of fatty fish and fish oil, resulting in higher vitamin D levels.

Traditionally, young children, pregnant women, and the elderly are considered at risk for vitamin D deficiency, although recent studies have also included adolescents and young adults as at-risk groups.^6^ Similarly, we observed that participants aged 18 to 39 years had more severe 25OHD status than those >49 years. This may be explained by the fact that young adults are under pressure from school or work, and therefore, spend large amounts of time indoors. In contrast, elderly Chinese individuals are mindful of their health and often exercise outdoors before and after work, thereby increasing their sunshine exposure. Interestingly, males in Beijing aged 49 to 59 years (who are at the peak of their career) had significantly lower 25OHD level. Although the cause of this reduction is not well understood, it might be attributed to smog level or professional pressures reducing these participants’ exposure to sunshine.

Regarding participant characteristics, earlier studies have reported that BMI is inversely associated with 25OHD,^7,28^ whereas we observed a positive association, although it was not significant after correcting for age and sex. Previous studies have also reported that vitamin D sufficiency positively impacts intestinal absorption of Ca and P,^10,29^ and we also observed a positive correlation between serum 25OHD and Ca levels. Traditionally, 25OHD can increase the absorption of P.^10,29^ In our study, P had a significant negative correlation with 25OHD (*r* = −0.095, *P* < 0.01). However, after correcting for age and sex, no significant correlation was observed between 25OHD and P. This result is different from some previous findings.^10,29^ Several other studies have reported an inverse association between iPTH and 25OHD level,^16,17,26^ which we also observed, even after correcting for age and sex. This relationship was also observed in the 117 participants with 25OHD >30 ng/mL, although the low number of participants precluded a more thorough analysis of the correlation between high 25OHD and iPTH level.

One limitation of this study was that the LC-MS/MS method we used could not separately evaluate epimers of 25OHD. However, 3-epi 25OHD_3_ is thought to have a negligible effect on routine LC-MS/MS testing for vitamin D, given its low concentration (<5 ng/mL)^30^ or complete absence in adult participants.^31^ To avoid overestimation, we compared our chromatography column [ACQUITY UPLC BEH (Phenomenex, Inc. Torrance, CA, US) Phenyl column] to a Phenomenex Kinetex PFP column that is capable of baseline separation of 25OHD_3_ and 3-epi 25OHD_3_ (Supplementary Figure 2, http://links.lww.com/MD/A218). Over a runtime of 13 minutes, we analyzed 307 samples using both methods and observed a linear correlation of 0.99 (all samples had <3 ng/mL 3-epi 25OHD_3_) with no significant difference between the 2 methods (Supplementary Figure 3, http://links.lww.com/MD/A218). Therefore, we believe that the data produced in this study is reliable and accurate. Another significant limitation of this study is that our results were obtained during summer, when participants are exposed to a maximum amount of sunshine. As sunshine exposure is the most important source of vitamin D,^10^ our observed vitamin D levels might be higher than the average levels throughout the year.

In conclusion, 25OHD status among Chinese adults was analyzed with a reliable LC-MS/MS method. Only a small proportion of the population had detectable level of 25OHD_2_, and 55.9% and 94.6% of participants had serum 25OHD <20 and <30 ng/mL, respectively. The 25OHD status was much worse among women and younger participants (<39 years), and the degree of hypovitaminosis D was higher in inland regions than coastal regions. Vitamin D insufficiency or deficiency is common among Chinese adults, and the health effects should be studied in the future.

## ACKNOWLEDGMENT

The authors wish to thank Professor Kiyoshi Ichihara for organizing the International Federation of Clinical Chemistry (IFCC) Global Multicenter study of reference intervals in China, as our study population was a part of that program.
