# The High Prevalence of Hypovitaminosis D in China: A Multicenter Vitamin D Status Survey: Erratum

**DOI:** 10.1097/01.md.0000463679.39876.03

**Published:** 2015-03-20

In the article “The High Prevalence of Hypovitaminosis D in China: A Multicenter Vitamin D Status Survey”^1^, which appeared in Volume 94, Issue 8 of *Medicine*, several corrections were required regarding various manufacturer locations. The article has since been corrected online.
